# Plastic accommodation at homophase interfaces between nanotwinned and recrystallized grains in an austenitic duplex-microstructured steel

**DOI:** 10.1080/14686996.2016.1140302

**Published:** 2016-03-01

**Authors:** Iván Gutierrez-Urrutia, Fady Archie, Dierk Raabe, Feng-Kai Yan, Nai-Rong Tao, Ke Lu

**Affiliations:** ^a^ Microstructure Physics and Alloy Design, Max-Planck-Institut fűr Eisenforschung, Max-Planck Str. 1, D-40237Dűsseldorf, Germany; ^b^ Structural Materials Unit, Research Center for Strategic Materials, National Institute for Materials Science, Tsukuba, Ibaraki 305-0047, Japan; ^c^ Institute of Metal Research, Chinese Academy of Sciences, Shenyang National Laboratory for Materials Science, Shenyang110016, PRChina; ^d^ Herbert Gleiter Institute of Nanoscience, Nanjing University of Science & Technology, Nanjing210094, PRChina

## Abstract

The plastic co-deformation behavior at the homophase interfaces between the hard nanotwinned grain inclusions and the soft recrystallized matrix grains in a duplex-microstructured AISI 316L austenitic stainless steel is examined through the analysis of long-range orientation gradients within the matrix grains by electron backscatter diffraction and transmission electron microcopy. Our analysis reveals that the mechanical accommodation of homophase interfaces until a macroscopic strain of 22% is realized within a small area of soft grains (about four grains) adjacent to the homophase interface. The activation of deformation twinning in the first two grain layers results in the occurrence of a ‘hump’ in the orientation gradient profile. We ascribe this effect to the role of deformation twinning on the generation of geometrically necessary dislocations. The smooth profile of the orientation gradient amplitude within the first 10 grain layers indicates a gradual plastic accommodation of the homophase interfaces upon straining. As a consequence, damage nucleation at such interfaces is impeded, resulting in an enhanced ductility of the single phase duplex-microstructured steel.

## Graphical abstract

**Figure:**
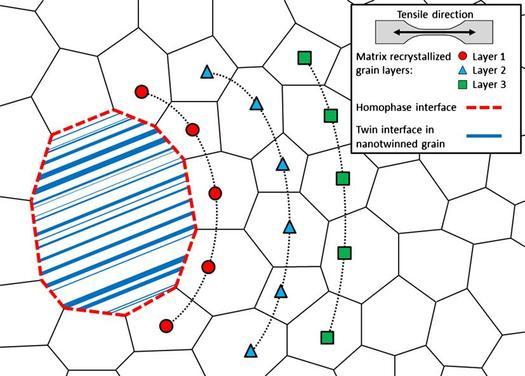

## 1. Introduction

Duplex-microstructured steels have been intensively investigated in the past years as a high performance class of advanced structural materials. These microstructures are commonly based on composite-type morphologies containing a hard phase, typically 20–30 vol.% martensite, and a soft phase, namely, ferrite [1, 3]. They are very attractive to the automotive industry because of their favorable combination of high strength and good formability [1,3,4]. The austenite to martensite transformation in these steel grades is accompanied by a 2–4% volume expansion, generating residual stresses and complex strain gradients in the matrix ferrite adjacent to the ferrite/martensite interface [5,6]. The residual stresses tend to enhance the plastic flow in ferrite and decrease the elastic limit while strain gradients accommodated by geometrically necessary dislocations (GNDs) contribute to the continuous yielding behavior that characterizes the strain-hardening behavior [5–9].

The large number of relevant microstructure parameters, the complexity of the underlying deformation mechanisms and the limited ductility when exposed to complex strain path changes make the design of these steel grades a challenging task [10–13]. Hence, recently, different approaches have been proposed to design single-phase heterogeneous structural steels containing homophase interfaces [14–21]. Among them, the processing strategies typically involve severe plastic deformation to high strain levels and subsequent annealing treatments to create fully or partially recrystallized microstructures containing different density/types of crystal defects or grain size distributions [14–19,22]. One of these approaches has successfully produced austenitic single phase duplex-microstructured steels consisting of coarse nanotwinned grains embedded into fine recrystallized matrix grains by means of dynamic plastic deformation (DPD) [17–19,23]. From a micromechanical standpoint, such microstructures can be considered as a single-phase composite consisting of hard inclusions, namely the nanotwinned grains, surrounded by softer recrystallized grains. Specifically, the tensile strength of nanotwinned grains can be as high as ~1.5–2.0 GPa, i.e. higher than martensite, but can yet sustain ~5% uniform tensile strain [18,19].

Homophase interfaces such as dislocation boundaries and twin interfaces play a significant role on the strain-hardening behavior, and hence on the mechanical properties of polycrystalline materials. The most evident interface parameter that contributes to the strain-hardening behavior of polycrystalline materials is the interface spacing. According to dislocation-mean free path theories of strain-hardening [24,25], homophase interface spacing contributes to the macroscopic flow stress by scaling laws such as the Hall-Petch relation and the similitude relation [26–29]. However, other homophase interface parameters have a significant contribution to the strain-hardening behavior as well: misorientation of the dislocation boundary and twin thickness determine the critical stress required to transfer plasticity across dislocation boundaries and twin interfaces, respectively [30,31]; elastic and plastic mismatch between the iso-phase control the plastic accommodation of the homophase interface, and hence the formation of strain gradients accommodated by geometrically necessary dislocations (GNDs) around such interfaces [32–34].

In a previous work [23], we have investigated the plastic deformation mechanisms of a novel austenitic duplex microstructured steel fabricated by dynamic plastic deformation (DPD). The duplex microstructure consists of strong nanotwinned grains embedded in soft recrystallized grains. We observed that at low strain levels (below 5%), the material deforms homogeneously by gradual co-deformation between the hard and soft grains without producing noticeable strain localization at the homophase interfaces between the two types of grains. With further straining (over 10%), a strain gradient is developed within the softer grains as a function of the distance from the homophase interfaces. This effect, together with the activation of localized deformation in the form of shear banding within the coarse nanotwinned grains, results in an inhomogeneous deformation behavior of the duplex steel. It is thus clear that the mechanical behavior of the homophase interfaces between the hard and soft grains plays a significant role on the deformation behavior, and hence, on the mechanical behavior of this advanced steel.

The present work investigates the details of the plastic accommodation of homophase interfaces through the analysis of the evolving long-range orientation gradients within the recrystallized matrix grains by electron backscatter diffraction (EBSD) and transmission electron microscopy (TEM). Our analysis of in-grain orientation gradients reveals that the mechanical accommodation of homophase interfaces until a macroscopic strain of 22% is realized within a small area of soft grains (about four grains) adjacent to such interface. The activation of deformation twinning in the first two soft grain layers close to the homophase interface results in the occurrence of a ‘hump’ in the orientation gradient profile. We ascribe this effect to the role of deformation twinning on the generation of geometrically necessary dislocations. The smooth profile of the orientation gradient amplitude within the first 10 grain layers indicates a gradual plastic accommodation of such interface upon straining. We associate this finding to the good ductility exhibited by the present steel (total elongation of about 46%).

## 2. Experimental methodology

**Figure 1.**
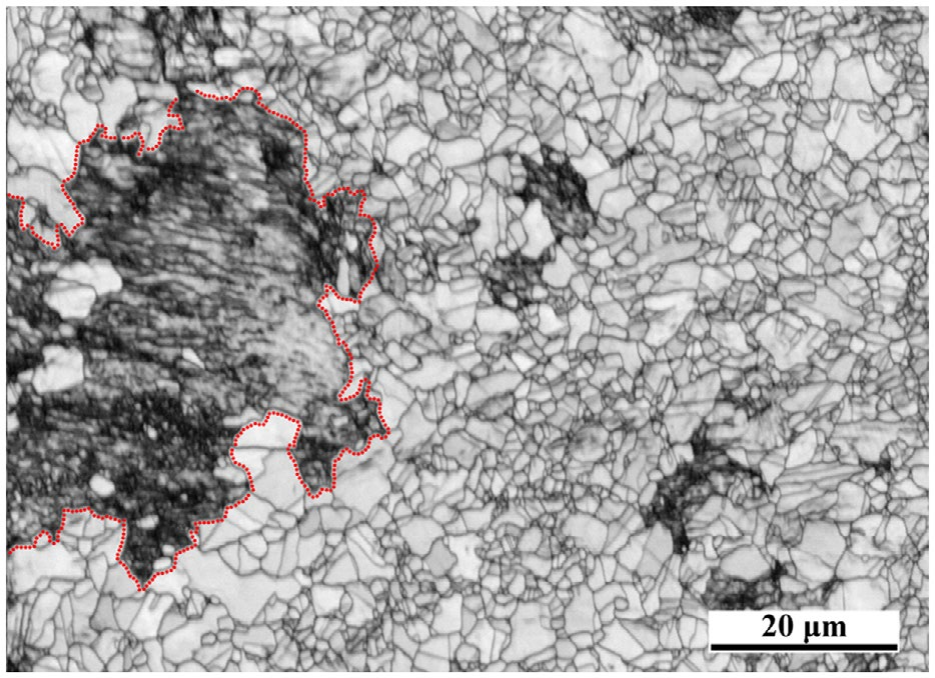
EBSD map of the duplex microstructured AISI 316L stainless steel consisting of coarse nanotwinned grains and fine recrystallized grains. The homophase between the two grain types is indicated by a red dotted line.

The material used in this work was an AISI 316L austenitic single phase-duplex microstructured steel. The duplex microstructure consists of coarse inclusion grains (average grain size of 20 μm) with an area fraction of ~23%, and fine recrystallized matrix grains with an average grain size of ~2 μm (Figure 1). Most of the coarse grains contain a lamellar twin structure of nano-scale twins (thickness ~23 nm) arranged into bundles. The coarse twinned grains are considered as a hard inclusion phase and the recrystallized grains act as a soft matrix phase [19,23]. The duplex microstructure was produced via a two-step process, namely, imposing first a dynamic plastic deformation (DPD) processing through multiple impacts to a total strain of 1.6, and a subsequent annealing step at 750°C for 45 min [17,19,35]. Interrupted tensile tests to engineering strain levels of *ε*=0.05, 0.12 and 0.22 were performed to investigate the evolution of the deformation behavior of the duplex microstructured steel. The tensile samples had 5 mm gage length, 2 mm gage width and 1 mm gage thickness. The monotonic tensile deformation experiments were carried out on a tensile Kammrath & Weiss GmbH test instrument (44141 Dortmund, Germany) equipped with a digital image correlation (DIC) system (ARAMIS system, GOM-Gesellschaft für Optische Messtechnik mbH, 38106 Braunschweig, Germany) to measure the local and the macroscopic strain distribution [36]. The surface pattern required for DIC was obtained by applying two different color sprays on the sample surface. First, a white spray was used to obtain a homogeneous background, and then a black spray was applied to obtain a spotted pattern [37]. Averaged engineering strain values were retrieved from the corresponding strain maps. At each strain level, microstructure was characterized by EBSD after DIC surface pattern removal. The observation direction was parallel to the tensile axis. EBSD maps were acquired with a 6500 F JEOL field emission gun-scanning electron microscope (FEGSEM) equipped with a TSL OIM EBSD system at 15 kV acceleration voltage, working distance of 15 mm and step size of 50 nm. The details of TEM characterization are described in [23].

## 3. Results and discussion

### 3.1. Analysis method of orientation gradients

**Figure 2.**
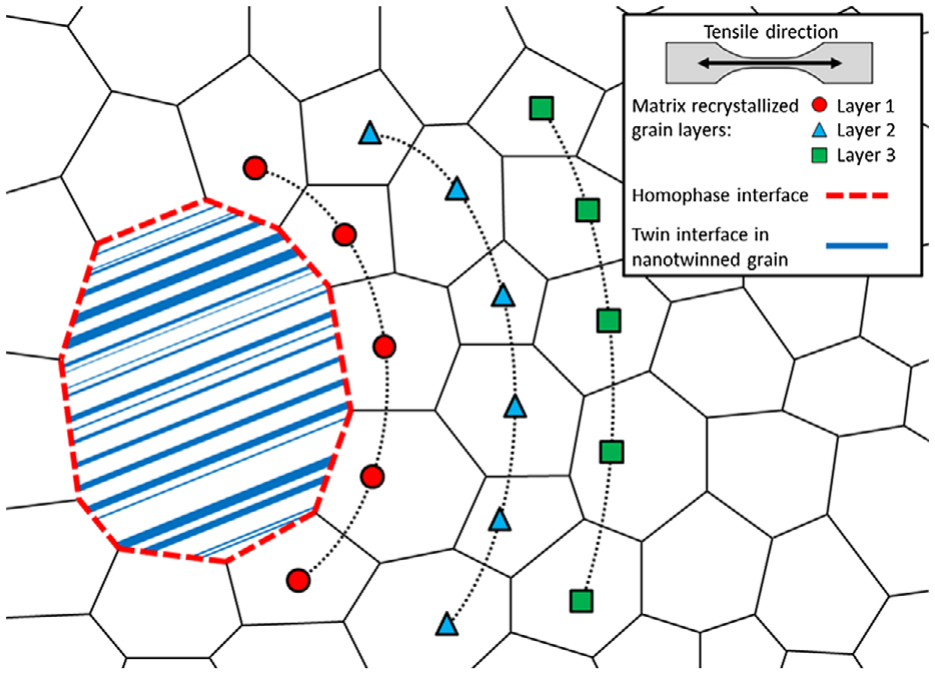
Sketch of the duplex-microstructure at the homophase interface region. The classification of matrix grains surrounding the hard grain inclusion into layers is depicted.

**Figure 3.**
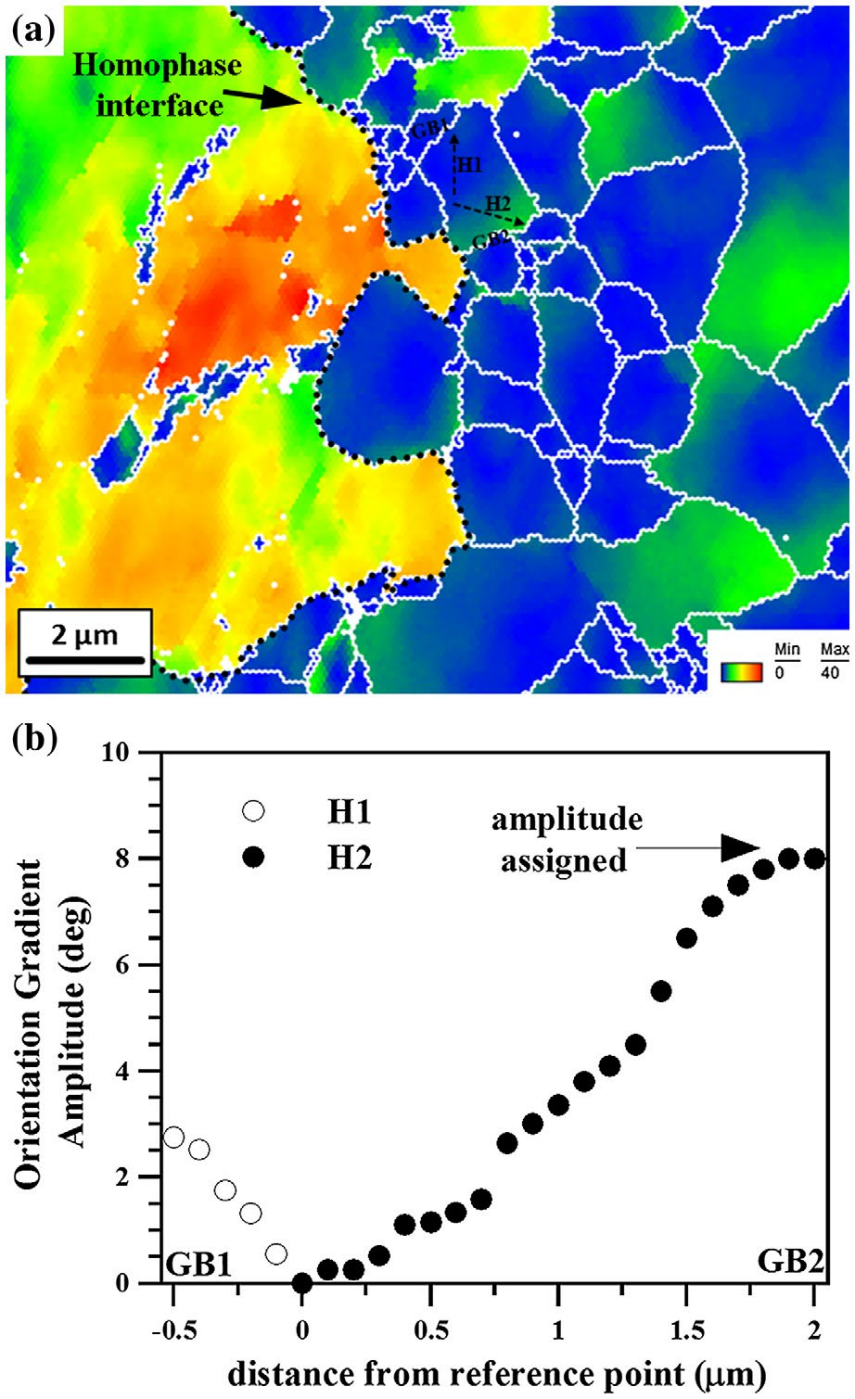
Sketch of the analysis of orientation gradients. (a) Example of grain reference orientation deviation (GROD) map. H1 and H2 refer to in-grain orientation gradients around grain boundaries labeled as GB1 and GB2, respectively. (b) Schematic plot of the amplitude of the orientation gradients shown in (a).

We investigated the deformation behavior in the nanotwinned grain/recrystallized grain (nt-grain/Rx-grain) interface regions by means of an EBSD approach based upon grain reference orientation deviation (GROD) maps. The method is outlined below in more detail. The deformation behavior in the homophase interface regions was evaluated along several matrix Rx-grain layers surrounding the hard inclusion grains, as depicted schematically in Figure 2. We have only considered areas containing similar grain sizes of the recrystallized matrix grains of about 2–5 μm to avoid blurring of the results due to grain size effects. The approach chosen here allows obtaining a sound microstructure–orientation gradient correlation for such heterostructure homophase morphologies in the present alloys. The rationale behind this method is to quantify the degree of strain localization near the interface of the nanotwinned grain inclusions. The evaluation method is as follows. First, the matrix Rx-grains surrounding a hard inclusion grain are classified into several layers according to their distance to the nearest homophase interface (Figure 2). Second, grain reference orientation deviation (GROD) maps are calculated from the EBSD data as a function of the angular deviation from a reference orientation within a given grain. Basically, GROD maps display in-grain misorientations with respect to the selected reference orientation. In the present case, we have set the reference orientation as the one containing the lowest Kernel average misorientation (KAM) value. This parameter is calculated as the average misorientation, $\Delta {\text{g}}_{\text{K}}$, of a given point relative to its neighbors, with the exclusion of misorientation values $\Delta {\text{g}}_{\text{Ai}\;}$that exceed a maximum tolerance value of 2° [38,39]:

[1] $$\Delta {\text{g}}_{\text{K}}=\frac{1}{4}\left(\Delta {\text{g}}_{\text{A}1}+\Delta {\text{g}}_{\text{A}2}+\Delta {\text{g}}_{\text{A}3}+\Delta {\text{g}}_{\text{A}4}\right)$$

where $\Delta {\text{g}}_{\text{Ai}}$ refers to the misorientation between a given point A and the neighbor i. Figure 3(a) shows an example of a GROD map corresponding to a matrix Rx-grain close to a homophase interface. This figure reveals that plastic deformation is concentrated at the grain boundaries rather than at the grain interiors, as reflected by the high local misorientation values. This effect can be ascribed to elastic and plastic incompatibility effects between neighboring grains which promote the activation of a higher number of slip systems compared to the grain interiors [40,41]. Figure 3(a) also reveals the development of several regions with high localized in-grain orientation gradients, namely H1 and H2, as expected in polycrystal plasticity owing to different boundary conditions and, hence, different accommodation gradients on opposite sides of the same grain [42,43]. As a general measure to quantify such in-grain orientation gradients in the different matrix Rx-grain layers around the homophase interface, we assign to each grain the orientation gradient amplitude with the highest GROD value, for instance the amplitude H2 in Figure 3(a), as depicted in Figure 3(b). We then calculate the average orientation gradient amplitude for each grain layer (about 10 grains per layer) and analyze them as a function of progressing sample deformation (the same grains are analyzed in each layer at evolving strain level).

### 3.2. Microstructure characterization

**Figure 4.**
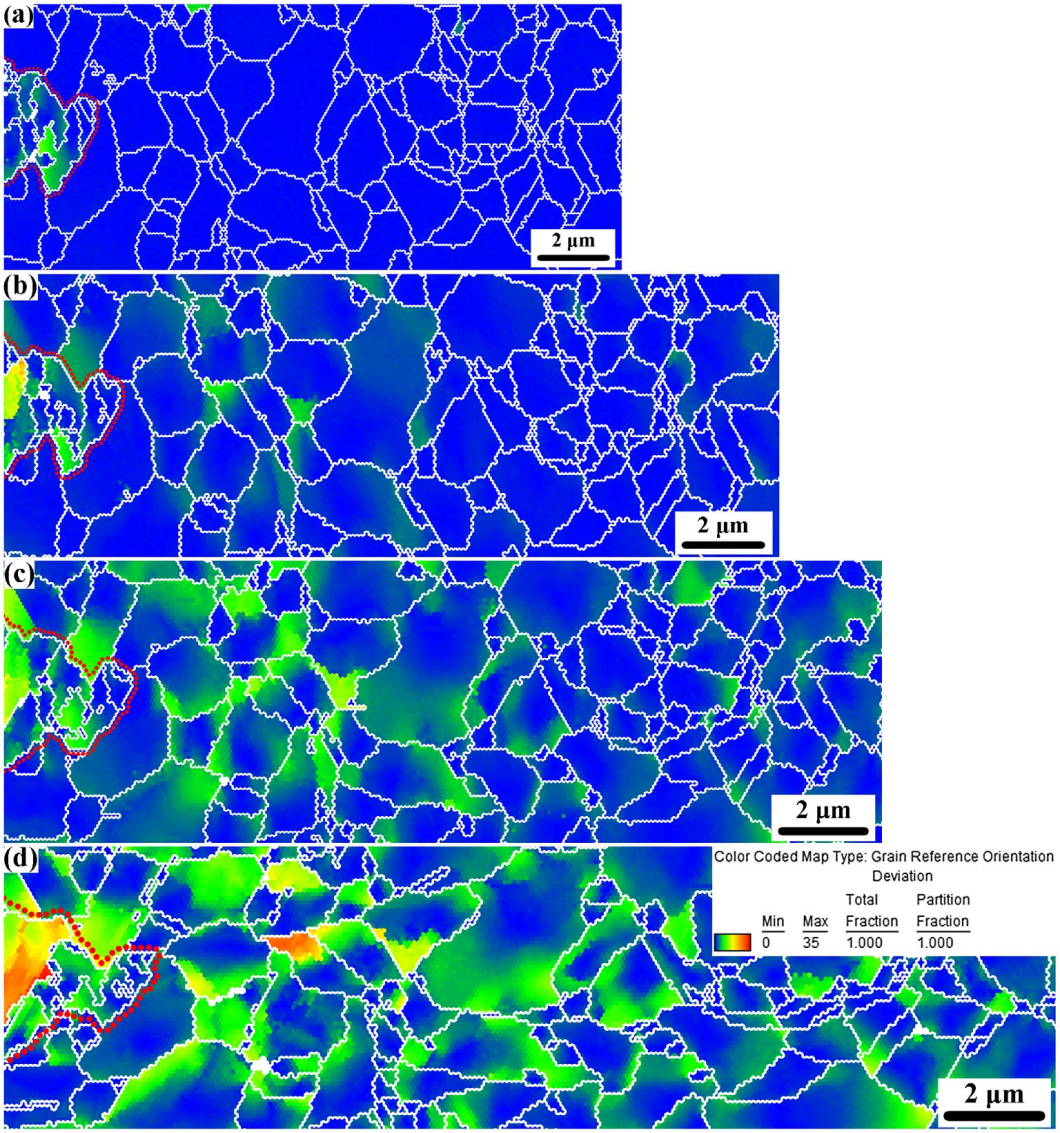
Grain reference orientation deviation (GROD) maps of the tracked duplex microstructure at several strain levels [23]. The homophase interface is indicated by a red dotted line. (a) undeformed; (b) engineering strain of 0.05; (c) 0.12; (d) 0.22.

As Figure 1 reveals, the microstructure of the AISI 316L austenitic single phase-duplex microstructured steel can be considered as a partially recrystallized composite-type structure formed by micron-sized Rx austenitic grains containing hard austenitic inclusions (outlined, non-recrystallized grains, i.e. mainly nt-grains). Figure 4 shows the corresponding GROD maps of the tracked area at several engineering strain levels [23]. These maps show the formation of local orientation gradients within the matrix Rx-grains, i.e. the soft phase, close to the homophase interface (indicated by a red dotted line in Figure 4) upon macroscopic straining. In the as-processed state, the coarse nt-grain (outlined by a red dotted line) contains orientation gradients which are ascribed to the high density of crystal defects whereas the Rx-grains are initially free of orientation gradients; see Figure 4(a). Orientation gradients within Rx-grains adjacent to the homophase interface are visible at a strain of 0.12; see Figure 4(c). Further straining to 0.22 engineering strain results in a similar distribution of orientation gradients within all the Rx-grains; see Figure 4(d).

**Figure 5.**
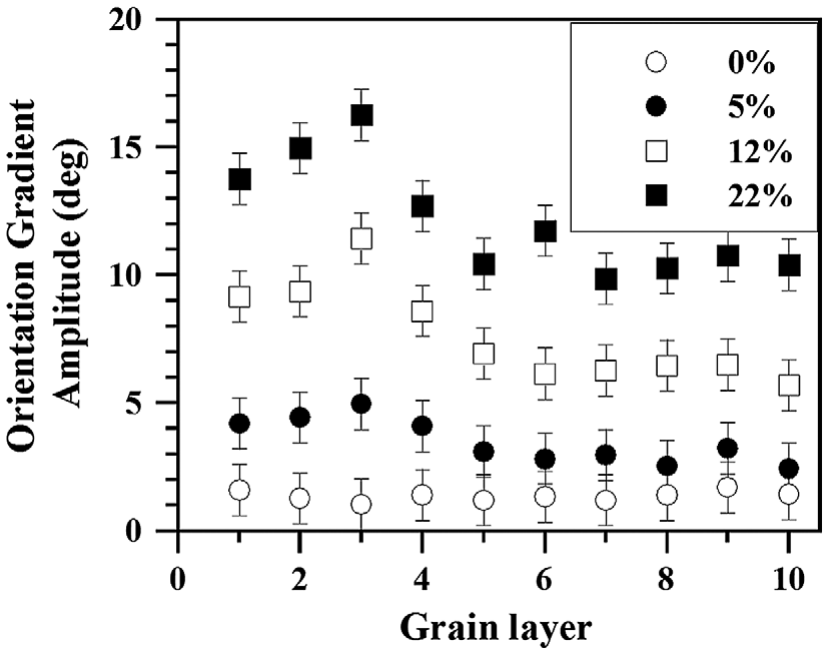
Evolution of the average orientation gradient amplitude with the imposed engineering strain along 10 matrix grain layers adjacent to the homophase interface shown in Figure 4. Error bars indicate statistical and experimental angular error (± 1°).

The evolution of the average orientation gradient amplitude as a function of the imposed macroscopic strain in the 10 matrix Rx-grain layers adjacent to the homophase interface of Figure 4 is shown in Figure 5. The main characteristics of this analysis are: first, we observe the development of a gradual transition from strong orientation gradients that are located within the first four grain layers to small orientation gradients occurring in grains further away from the immediate homophase interface. The intensity of the strong orientation gradients is about 1.5 times higher than the grains farther away from the interface. Second, we observe the occurrence of a ‘hump’ in the orientation gradient profile located at about the third grain layer. These observations indicate that the homophase interface plays a significant role on the deformation behavior of the nearest four grain layers to such interface. The development of an area of soft matrix Rx-grains with enhanced plastic activity, i.e. high density of geometrically necessary dislocations (GNDs), around a homophase interface can be ascribed to the mechanical incompatibility between the hard nt-grain and the soft Rx-grains resulting in a load transfer effect similar to that occurring in metal matrix composites [44] and second phase-particle containing materials [32–34]. The present results indicate that such mechanical incompatibility is mainly relaxed within the soft Rx-grains by the occurrence of strain gradients extending over about four grains.

**Figure 6.**
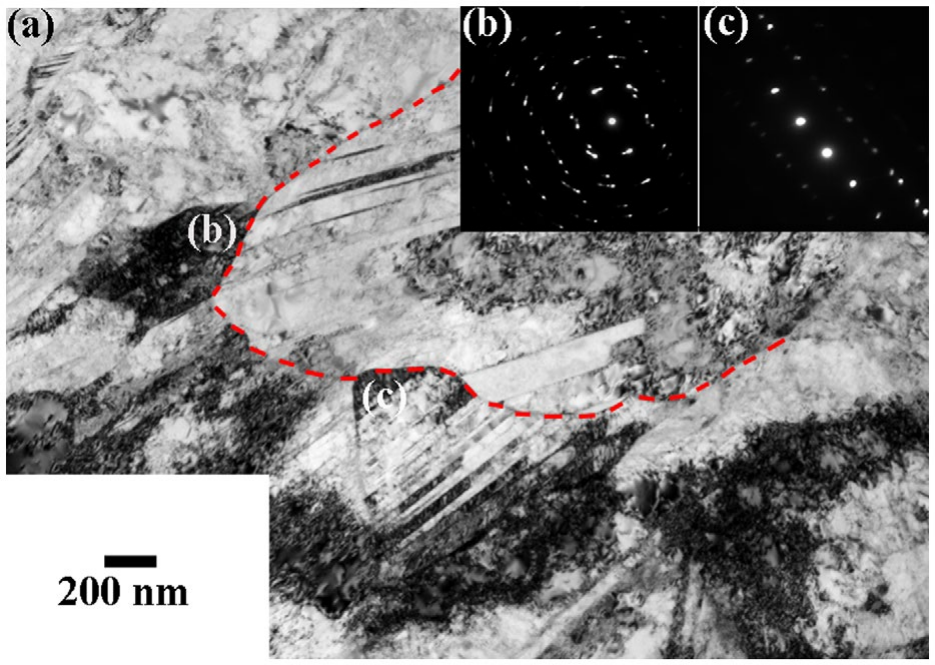
(a) Bright-field TEM image of the deformation structure of an area containing a single coarse nanotwinned grain (indicated by a red dotted line) and several adjacent recrystallized matrix grains. The sample was deformed in tension to a strain of 0.12. (b, c) Corresponding diffraction patterns of recrystallized matrix grains labeled as b and c in (a).

The occurrence of a ‘hump’ in the profile of the average orientation gradient amplitude deviates from the typical trend 1/*λ*, where *λ* is the distance from a given interface, reported in several prior studies [5,6,34]. Figure 5 also shows that the height of the ‘hump’ scales with strain. This observation suggests the occurrence of a governing relation between the evolution of the deformation substructure and the development of orientation gradients within the first four matrix Rx-grain layers around the homophase interface. TEM observations revealed a pronounced activation of deformation twins within the Rx-grains. As Figure 6 shows, at a macroscopic strain of 0.12, Rx-grains adjacent to the homophase interface, namely grains labeled as (a) and (b), develop a lamellar twin-type structure, as shown in the corresponding diffraction patterns. In contrast, Rx-grains located away from such homophase interfaces contain dislocation substructures free of deformation twins. As a previous work has recently shown [23, figures 1(b), 5(a) and 8], in the present single phase-duplex microstructured steel strained to low strain levels (below 5%), the homophase interfaces between the nanotwinned grains and the recrystallized grains are accommodated by dislocation plasticity. The nt-grains deform in a homogeneous fashion in conjunction with the surrounding Rx-grains without generating significant strain localization near their interfaces, as revealed by the homogeneous dislocation density distribution within the Rx-grains adjacent to such interfaces [23, figure 5(b)].

Deformation twinning in fcc steels is a stress-assisted mechanism which is dependent on the crystallographic grain orientation through the Schmid factor, and the grain size [31,37,45]. In the present composite-structure, we do not observe any preferential crystallographic orientation for twinning of the matrix Rx-grain layers. EBSD mapping reveals that these grains develop a typical α-fiber with texture components oriented along the line between the <001>//TA and <111>//TA crystallographic directions (TA: tensile axis). As these grains have similar average grain sizes, this lack of preferential crystallographic orientation suggests that the activation of deformation twinning in these grains is controlled by the local stress state, which can strongly vary from the macroscopic stress state [37,45]. Such local high stress concentrations result from the plastic strain mismatch between the stiff nt-grain and the soft Rx-grain.

The occurrence of a ‘hump’ in the profile of the average orientation gradient amplitude can be therefore explained as follows. Orientation gradients in second-phase containing materials are determined by the generation of geometrically necessary dislocations, *ρ*_*GND*_, required to accommodate the plastic gradient ascribed to the mechanical incompatibility between the hard inclusion and the soft matrix. This effect can be roughly estimated as *ρ*_*GND*_ ~ *ε/λ*, where *λ* is the distance from a homophase interface and *ε* is the difference of plastic strain between the soft phase and hard phase [33,34]. However, the activation of deformation twinning modifies this picture. TEM observations in the present material reveal that at a macroscopic strain of 0.12–0.22 twins are mainly visible within the first and second Rx-grains adjacent to the homophase interface [23]. These observations indicate that grain deformation can be carried out in these grains by slip and twinning. We suggest that the competition between these two deformation modes to accommodate the plastic gradient associated to the mechanical incompatibility between the hard inclusion and the soft matrix, as well as slip hardening due to twin-slip interaction result in smaller *ρ*_*GND*_ compared to the case where only slip is available, such in Rx-grains away from the homophase interface. As a consequence, the orientation gradient profile deviates from a simple trend ~ 1/*λ* but exhibits a more complicated behavior.

At this point, it is also relevant to discuss the role of crystallographic grain orientation on slip transfer, and hence, on the occurrence of local orientation gradients. The geometry of slip transfer between two slip systems on either side of a boundary is usually defined by three angles, namely, the angle between slip vectors (κ), the angle between slip plane normals (ψ), and the angle between the two slip plane intersections with the grain boundary plane (φ) [46]. So far, two criteria have been proposed, which are based on the maximizing of a parameter that is a product of the cosine of some of these angles. Following these criteria, grain boundaries can be classified as impenetrable, penetrable and transparent according to their ability to transfer an incoming slip system. In the present work, we have investigated the occurrence of in-grain orientation gradients with respect to the distance of a specific homophase interface by EBSD. We define such distance as grain layers (up to 10), which are defined as grain neighbors to the interface. We then calculate the orientation gradient amplitude of each grain layer as the average amplitude of the highest GROD amplitude of 10 grains. In other words, the orientation gradient amplitude plotted in Figure 5 corresponds to the average of the orientation gradient amplitude of 10 grains per layer. Taking into account that the crystallographic orientations of these grains are contained within an α-fiber, i.e. they contain discrete grain orientations, and the intrinsic EBSD resolution (∼1–2° [47]), we consider that grain orientation effects on slip transfer, and hence on the amplitude of in-grain orientation gradients, are smoothed out in the present analysis.

It is important to recognize the excellent mechanical compatibility exhibited by the present homophase interface between the hard nanotwinned grain inclusion and soft Rx-grains. The present results reveal that the accommodation of the mechanical incompatibility between the two grain types is carried out in a gradual fashion both by slip and twinning along the four adjacent matrix Rx-grains to the interface, as reflected by the smooth profile of the orientation gradient amplitude. This result has a pronounced effect in attaining a high ductility (total elongation of the present single phase duplex-microstructured steel is about 46%). Both experimental and computational studies report that damage in particle-free materials commonly nucleates at locations of large strain incompatibilities such as grain or phase boundaries where high heterogeneous strain gradients can be developed [41,43,46]. Specifically, damage nucleation is dependent on several aspects such as the boundary orientation and structure, the grain boundary slip transfer geometry, i.e. slip/twin planes and directions of active deformation systems on either side of the boundary, and the stress–strain gradient history in the grains on either side of an interface [41,43,46,48,49]. The development of long-scale heterogeneous strain gradients at such homophase interfaces may cause large tensile tractions in the boundary resulting in damage nucleation [48,50]. The present analysis suggests that the gradual accommodation of the plastic deformation within the homophase interface region mitigates damage nucleation at such interfaces, and accordingly enhances the ductility of the duplex-microstructured steel.

## 5. Conclusions

We have investigated the plastic co-deformation behavior of an austenitic duplex-microstructured AISI 316L stainless steel at the homophase interfaces between hard nanotwinned grain inclusions and soft recrystallized matrix grains. The evolution of the underlying deformation structure as a function of strain was investigated by using EBSD and TEM. In-grain orientation gradients along the recrystallized matrix grains are analyzed by means of a grain reference orientation deviation-type approach based on EBSD data. The following conclusions can be drawn:

• Our analysis of in-grain orientation gradients reveals that the mechanical accommodation of homophase interfaces until a macroscopic strain of 22% is realized within a small area of soft grains (about four grains) adjacent to the homophase interface. The activation of deformation twinning in the first two soft grain layers close to the homophase interfaces results in the occurrence of a ‘hump’ in the orientation gradient profile. We ascribe this effect to the role of deformation twinning on the generation of geometrically necessary dislocations.
• The smooth profile of the orientation gradient amplitude within the first 10 Rx-grain layers indicates that the mechanical accommodation of the interface is realized in a gradual fashion along the adjacent matrix grains surrounding the hard inclusion grains.
• We ascribe the good ductility exhibited by the present austenitic duplex-microstructured steel (total elongation of about 46%) to the gradual accommodation of plastic deformation by multiple slip system activation within the homophase interface region that leads to GNDs, which hinders the occurrence of high local orientation gradients that can nucleate damage at such interfaces, and accordingly enhances the total elongation.

## Disclosure statement

No potential conflict of interest was reported by the authors.
